# Curcumin Ameliorates Nonalcoholic Fatty Liver Disease through Inhibition of *O*-GlcNAcylation

**DOI:** 10.3390/nu11112702

**Published:** 2019-11-08

**Authors:** Da Eun Lee, Su Jin Lee, Su Ji Kim, Hyun-Shik Lee, Oh-Shin Kwon

**Affiliations:** School of Life Science and Biotechnology, BK21 Plus KNU Creative BioResearch Group, College of Natural Science, Kyungpook National University, Daegu 41566, Korea; starkr0@hanmail.net (D.E.L.); neojove79@naver.com (S.J.L.); susie514@hanmail.net (S.J.K.); leeh@knu.ac.kr (H.-S.L.)

**Keywords:** liver, *O*-GlcNAcylation, curcumin, NAFLD and SOD1

## Abstract

The cause of progression to non-alcoholic fatty liver disease (NAFLD) is not fully understood. In the present study, we aimed to investigate how curcumin, a natural phytopolyphenol pigment, ameliorates NAFLD. Initially, we demonstrated that curcumin dramatically suppresses fat accumulation and hepatic injury induced in methionine and choline-deficient (MCD) diet mice. The severity of hepatic inflammation was alleviated by curcumin treatment. To identify the proteins involved in the pathogenesis of NAFLD, we also characterized the hepatic proteome in MCD diet mice. As a result of two-dimensional proteomic analysis, it was confirmed that thirteen proteins including antioxidant protein were differentially expressed in hepatic steatosis. However, the difference in expression was markedly improved by curcumin treatment. Interestingly, eight of the identified proteins are known to undergo *O*-GlcNAcylation modification. Thus, we further focused on elucidating how the regulation of *O*-linked β-*N*-acetylglucosamine (*O*-GlcNAc) modification is associated with the progression of hepatic steatosis leading to hepatitis in MCD diet mice. In parallel with lipid accumulation and inflammation, the MCD diet significantly up-regulated hexosamine biosynthetic pathway (HBP) and *O*-GlcNAc transferase (OGT) via ER stress. Curcumin treatment alleviates the severity of hepatic steatosis by relieving the dependence of *O*-GlcNAcylation on nuclear factor-κB (NF-κB) in inflammation signaling. Conversely, the expressions of superoxide dismutase 1 (SOD1) and SIRT1 were significantly upregulated by curcumin treatment. In conclusion, curcumin inhibits *O*-GlcNAcylation pathway, leading to antioxidant responses in non-alcoholic steatohepatitis (NASH) mice. Therefore, curcumin will be a promising therapeutic agent for diseases involving hyper-*O*-GlcNAcylation, including cancer.

## 1. Introduction

The etiology of non-alcoholic fatty liver disease (NAFLD) is poorly understood. It is believed to be initiated by the accumulation of lipids in the liver and then may progress to non-alcoholic steatohepatitis (NASH) involving inflammation and fibrosis [1,2]. The first characteristic histological lesions of NAFLD is the abnormal accumulation of lipids in hepatocytes [3]. For free fatty acids (FFA) accumulation, there are several mediating molecules such as peroxisome proliferator activating ligand receptors (PPARs), liver X receptor (LXR), and AMP-activated protein kinase (AMPK) [4]. The second characteristic feature of NAFLD is increased lipid peroxidation and oxidative stress [5,6]. Oxidative stress, particularly lipid peroxidation, occurs as a result of excessive reactive oxygen species (ROS) production or reduced antioxidant defenses in hepatocytes, leading to liver damage and the activation of hepatic stellate cells [7,8]. Antioxidant enzymes play an important role in regulating oxidative stress in cells. Superoxide dismutase 1 (SOD1) is a major antioxidant enzyme and is mainly present in the cytoplasm and mitochondria. It can catalyze the conversion of peroxide anions to H_2_O_2_, which is removed by catalase (CAT) [9,10]. Another antioxidant enzyme, glutathione peroxidase (GPx), further promotes the conversion of H_2_O_2_ to H_2_O [11]. SIRT1, a NAD-dependent deacetylase, is another key factor in regulation of inflammation and antioxidant defense systems [12]. SIRT1 (number of the sirtuin family) plays an important role in lipid and glucose homeostasis and insulin sensitivity by regulating mitochondrial viability and β-oxidation and improving anti-inflammatory activity [13]. It has been reported that there is a decrease in the activity of SOD1, GPx and CAT and a decrease in SIRT1 levels at the onset of NAFLD [14].

Lipotoxicity causes ROS accumulation and related endoplasmic reticulum (ER) stresses [15,16]. IRE1α (inositol requiring enzyme 1α), a downstream target gene of X-box binding protein 1 (XBP1) induced by fat toxicity, activates the proinflammatory transcription factor nuclear factor-κB (NF-κB) pathway, resulting in liver inflammation, which in turn promotes NASH [17,18]. XBP1 is involved as a key transcription factor in hepatic lipogenesis and inflammatory processes through ER stress [19]. Thus, this is another evidence suggesting that lipid pathways and inflammatory processes are closely linked. In addition, the accumulation of triglycerides in the hepatocyte appears to play a direct role in hepatocellular injury and inflammation, resulting in the production of ROS and cytokines such as tumor necrosis factor-α (TNF-α), interleukins (IL-1 and IL-6). After all, these processes activate hepatic stellate cells (HSCs), leading to pro-inflammatory and pro-fibrogenic functions [20,21]. NF-κB may critically be associated with many pathogenic liver conditions [22]. When stimulated with FFA, Toll-like receptor 4 (TLR4) activates MyD88-dependent signaling and induces NF-κB activation, causing the production of all inflammatory cytokines, including TNFα and IL6. The NF-κB pathway is regulated by ROS through various reversible post-translational modifications (PTMs) on the subunits, and more recently it has been reported that NF-κB activity is also regulated by *O*-linked β-*N*-acetylglucosamine (*O*-GlcNAc) [23].

*O*-GlcNAcylation is a reversible PTM involving the addition of *O*-GlcNAc on cytoplasmic and nuclear proteins [24]. Two intracellular enzymes, *O*-GlcNAc transferase (OGT) and *O*-GlcNAcase (OGA), regulate the level of *O*-GlcNAcylation on proteins [25]. OGT transfers the *O*-GlcNAc group onto serine or threonine residues on the target protein whereas OGA catalyzes the reverse reaction that removes *O*-GlcNAc from proteins. It is known that *O*-GlcNAcylation levels are regulated through the hexosamine biosynthetic pathway (HBP), in which the transcription factor XBP1, which regulates the immune system and ER stress responses, acts as an upstream regulator [26,27]. *O*-GlcNAcylation is an endpoint of HBP through UDP-GlcNAc production. *O*-GlcNAcylation modulates protein functions according to glucose availability and its flux through HBP [28]. In addition to glucose, *O*-GlcNAc also includes amine and acetyl moieties, suggesting that the availability of nutrients is sensed by this pathway. Recent reports have shown that infusion of a lipid emulsion increases the UDP-GlcNAc content in rats [29], and fatty acids directly up-regulate the expression of enzymes in HBP, including glutamine:fructose-6-phosphate amidotransferase (GFAT) [30]. Thus, blood glucose and fatty acid levels are likely to regulate numerous cellular processes, including inflammation, through protein *O*-GlcNAcylation.

Curcumin is a natural polyphenol compound derived from turmeric and exhibits two essential bioactivities in vitro and in vivo [31]. The first one is that curcumin exerts antioxidant activity via scavenging ROS [32]. This property makes curcumin hepatoprotective that helps prevent and resolve liver injuries. Several studies showed that curcumin may have a beneficial role in mitigating NASH development through its anti-inflammatory actions as well as restoring the balance of hepatic antioxidant systems. Secondly, curcumin acts as a Micheal acceptor and thus has potency as an anti-carcinogenic agent. These effects are related to the negative regulation of various transcription factors including NF-κB, PPAR-gamma, p53, and carbohydrate response element-binding protein ChREBP [33,34]. Curcumin also reportedly suppresses growth factors, cell proliferation factors and inflammatory cytokines, such as TNF-α, interleukins (IL-1 and IL-6) and cyclooxygenase-2 (COX-2) [35]. Although curcumin is known to have a variety of biological effects, the underlying mechanisms of its effect on NASH are largely unknown.

Thus, one aim of the present study was to confirm that curcumin ameliorates NAFLD by decreasing lipid accumulation and inflammation and improving the anti-oxidative status. In this study, we used methionine and choline-deficient (MCD) diet mice to characterize the liver proteome and identify proteins that are associated with steatohepatitis and have a high response to curcumin. The MCD diet mice have been proven as a useful experimental model for steatohepatitis [36,37]. The other aim was to elucidate how *O*-GlcNAcylation is related to NASH progression. Here, we demonstrated that curcumin restores aggravated liver metabolic damage by blocking the *O*-GlcNAc signaling pathway. Conclusively, inhibiting *O*-GlcNAcylation may be a promising approach to the treatment of NASH.

## 2. Materials and Methods

### 2.1. Animals

C57BL/6J male mice (seven weeks of age) were purchased from Hyochang Science (Daegu, Korea). All mice were housed in cages and maintained under 12 h light-dark cycle and conditioned to proper temperature (20–24 °C). Mice were decided into three experimental groups. The Control group was fed the normal diet (ND) for three weeks. The second group was fed the MCD diet for three weeks. The third group was the MCD diet, oral gavage with curcumin (100 mg/kg) once daily for three weeks. The animal experimental procedures were reviewed and approved by the animal care and use committee of the Kyungpook National University and were in compliance with the experimental animal regulation protocols of the Kyungpook National University, Korea.

### 2.2. Immunoblot Analysis

Hepatic liver tissue and AML12 cells were lysed with an NP40 buffer containing 150 mM NaCl, 50 mM HEPES (pH 7.4), 1 mM EDTA, 1% NP40, 2 mM sodium orthovanadate, and 1 μM okadaic acid before protein quantification with Coomassie Protein Assay Reagent (BioRad, Contra Costa County, CA, USA). Then, 25 μg of proteins were diluted in Laemmli sample buffer (final concentrations: 50 mM Tris (pH 6.8), 10% (*v*/*v*) glycerol, 2% sodium dodecyl sulfate (SDS) (*w*/*v*), and 0.01% (*w*/*v*) bromophenol blue] and separated by sodium dodecyl sulfate-polyacrylamide gel electrophoresis (SDS-PAGE) at 90–150 V, followed by transferring to nitrocellulose membranes (GE Healthcare Life science, Boston, MA, USA) at 100 V. The membranes were blocked with 5% *w*/*v* skim milk in Tris-base buffered saline-Tween 20 (TBS-T) for 1 h and probed by anti-goat primary antibody solution in TBS-T containing 0.02% (*w*/*v*) sodium azide for 2 h. Next, membranes were washed in TBS-T (3 × 10 min) and then probed with the appropriate horseradish peroxidase-coupled anti-goat secondary antibody (Cell Signaling, Danvers, MA, USA). After further washing in TBS-T, immunoprobed proteins were visualized using an enhanced chemiluminescence method (Visual Protein Co., Taipei, Taiwan) Antibodies against α-smooth muscle actin (α-SMA), *O*-GlcNAc (Sigma Aldrich, St. Louis, MO, USA), p-AMPKα, AMPKα, phosphorylated liver kinase B1 (p-LKB1), LKB1, phosphorylated Acetyl-CoA carboxylase (p-ACC), ACC, phosphorylated IκB kinase α/β (p-IKKα/β), IKKα, p-IκBα, p-NF-κB p65, IRE1α, OGT, SIRT1 (Cell signaling), Sterol regulatory element-binding protein (SREBP), fatty acid synthase (FAS), AnnexinA5 (ANXA5), peroxiredoxin 6 (Prx6), ChREBP, SOD1 (Santa Cruz, Dallas, TX, USA), Cytokeratin 8 (CK8), Cytokeratin 18 (CK18), and OGA (Abcam, Cambridge, MA, USA) were used as probes. The anti-actin antibody (Santa Cruz) was used as a loading control.

### 2.3. Immunohistochemistry (IHC) and Oil Red O Staining

Liver tissues were fixed in formalin and then embedded in paraffin. The slide section of liver tissue was stained with hematoxylin–eosin (H&E) and Oil Red O for hepatic histological analysis. Paraffin-embedded livers were cut into sections and stained with primary antibodies for protein localization. Epitope specific antibodies were used for ChREBP, α-SMA, CK18, SIRT1, OGT, F4/80 and SOD1 staining. Chromogenic detection was using with the DAB (DAKO). Then, the slides were dehydrated and mounted in Safemount embedding medium (Labonord, France).

For Oil Red O staining, frozen liver sections (10 μm) were fixed with 10% formaldehyde for 10 min. To measure lipid accumulation, slides containing the cryosections were washed in water, immersed in 100% propylene glycol for 5 min, and then incubated in 0.5% Oil Red O working solution (Sigma O0625) for 30 min at room temperature. The slides were washed with 85% propylene glycol (dilution in distilled water) for 20 min. Slides washed in distilled water for 5 min and then mounted in aqueous mounting medium (mixed gelatin, glycerol, and distilled water).

### 2.4. Quantitative Reverse Transcription–Polymerase Chain Reaction

Quantitative real-time (qRT) PCR analysis was performed using an established procedure [Lee et al., 2013]. Total RNA was isolated from the livers using the TRIzol reagent (Invitrogen, Carlsbad, CA, USA) and cDNA using QIAGEN extraction kits, and reverse transcription was performed using the SuperScript III RNase H reverse transcriptase kit (Invitrogen). Quantitative RT-PCR was performed using SYBR Green I and LightCycler (Roche Diagnostics, Castle Hill, Australia). For normalized gene expression, 18S rRNA was used as the reference gene in all qRT-PCRs. The following PCR primer sequences were used: 18S rRNA forward, 5′-GTA ACC CGT TGA ACC CCA TT-3′, 18S rRNA reverse, 5′-CCA TCC AAT CGG TAG TAG CG-3′, TNFα forward, 5′-CAC CAC CAT CAA GGA CTC TCA AA-3′, TNFα reverse, 5′-AGG CAA CCT GAC CAC TCT CC-3′, IL-6 forward, 5′-GAC AAC TTT GGC ATT GTG G-3′, IL-6 reverse, 5′-ATG CAG GGA TGA TGT TCT G-3′, TLR4 forward, 5ʹ-ACC TCT GCC TTC ACT ACA GA-3′, TLR4 reverse, 5′-AGG GAC TTC TCA ACC TTC TC-3′, XBP1 forward, 5′-AAG AAC ACG CTT GGG AAT GG-3′, XBP1 reverse, 5′-ACT CCC CTT GGC CTC CAC-3′, GFAT1 forward, 5′-TAA GGA GAT CCA GCG GTG TC-3′, and GFAT1 reverse, 5′-CAG CTG TCT CGC CTG ATT GA-3′. The results were normalized gene expression of 18S rRNA in all experiments. Subsequently, 50 ng of cDNA was used for qRT-PCR analysis, which was carried out with the StepOnePlus™ Real-Time PCR system (Applied Biosystems, Waltham, MA, USA).

### 2.5. Cell Culture and Transfection

The murine hepatocyte-derived AML12 cells were provided by Dr. Jae Man Lee (KNUM. Korea). The cell line was maintained in Dulbecco’s modified Eagle’s medium/Ham’s nutrient mixture F-12 containing 10% fetal bovine serum, 1% penicillin and 100 μg/L streptomycin at 37 °C in a 5% CO_2_ atmosphere. Control and SIRT1 siRNA (Santa Cruz) was transfected into AML12 cells using Lipofectamine RNAiMAX (Invitrogen). Cells were homogenized in NP40 buffer, and the immunoblot procedure was applied. The primary antibody used was SIRT1 for detecting proteins.

### 2.6. The ROS Production Assay

The ROS production in AML12 cells was detected using 2′,7′—dichlorofluorescin diacetate (DCFDA) (Invitrogen). AML12 cells were washed three times with phosphate buffered saline (PBS) and added to a Dulbecco’s Modified Eagle Medium (DMEM) containing 25 μM DCFDA. Cells were incubated for 20 min at 37 °C. Cells were washed three times again with PBS. Imaging was done by fluorescence microscopy (LEICA DMI3000 B, Wetzlar, Germany) using green filters.

### 2.7. Immunoprecipitation (IP)

Total liver lysates (200–500 μg) were incubated with antibody to p65 or ChREBP, for 12 h at 4 °C and using Dynabeads TM Protein A IP kit (Thermo Fisher Scientific, Waltham, MA, USA). Immunoprecipitated proteins were analyzed by boiling with 2X sample buffer. Protein samples were separated by SDS-PAGE and then electro-transferred to a membrane. After IP, immunoblot analysis was performed using antibodies against acetyl-lysine (Millipore, Billerica, MA, USA), p65 and ChREBP.

### 2.8. Statistical Analysis

Data are expressed as means ± SD of the indicated number of measurements. Data were analyzed using Student’s *t*-test. All statistical analyses were performed using the SPSS software (International Business Machines, Armonk, NY, USA), version 25.0 was considered statistically significant when *p*-value was less than 0.05.

## 3. Results

### 3.1. Effect of Curcumin on Hepatic Steatosis in MCD Diet Mice

**Figure 1:**
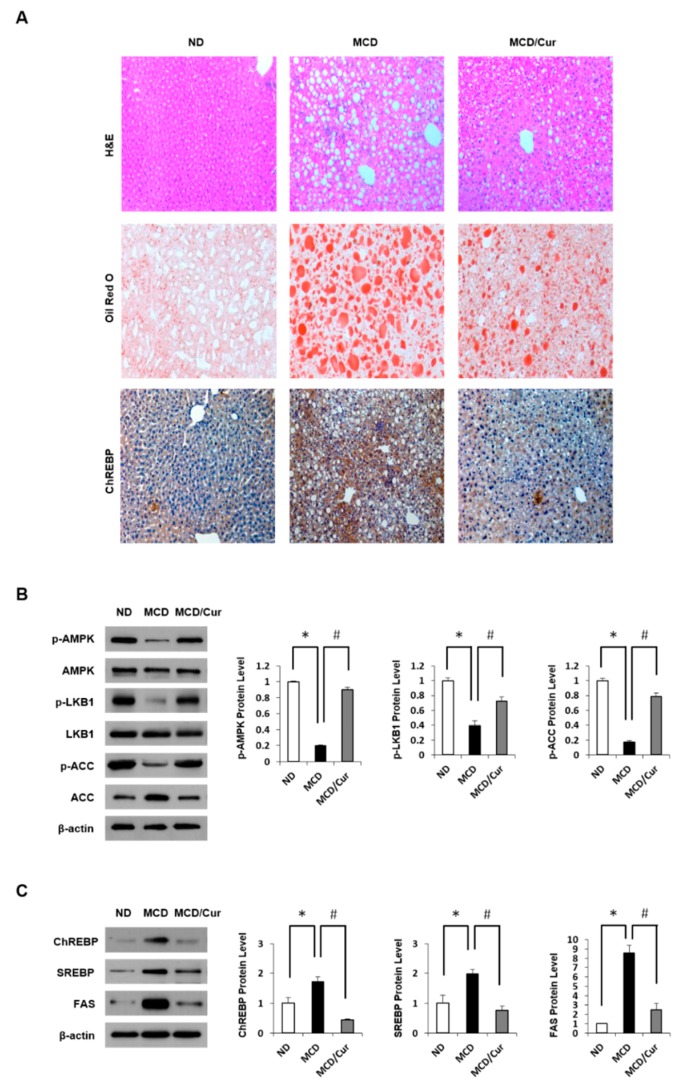
Effect of curcumin on lipid accumulation in methionine and choline-deficient (MCD) diet mice. Mice were fed a normal diet (ND), MCD diet, or MCD/curcumin (100 mg/kg) diet for 3 weeks. (**A**) Representative pictures of liver sections from these groups were assessed by hematoxylin-eosin (H&E) staining, Oil Red O staining, and immunohistochemical (IHC) analysis against carbohydrate response element-binding protein (ChREBP) (original magnification 200×). (**B**) and (**C**), Hepatic expression levels of phosphorylated AMP-activated protein kinase (p-AMPK), phosphorylated Liver kinase B1 (p-LKB1), and phosphorylated Acetyl-CoA carboxylase (p-ACC) along with ChREBP, Sterol regulatory element-binding protein (SREBP) and Fatty acid synthase (FAS) were measured by immunoblot assay. The levels of proteins were normalized to endogenous β-actin protein for each group. Data are mean ± SD (*n* = 7/group). * *p* < 0.05 vs. ND diet group, and ^#^ *p* < 0.05 vs. MCD diet group.

In previous studies, liver histology of MCD diet mice revealed macrovesicular lipid accumulation and moderate lobular inflammation, similar to the observation in human cases of NASH [36]. Feeding MCD diet to mice for three weeks increased in progressive weight loss and hepatic triglyceride (TG) concentration (Figure S1A). Moreover, serum alanine aminotransferase (ALT) and aspartate aminotransferase (AST) levels were markedly elevated in MCD diet mice compared with ND mice (Figure S1B). However, curcumin treatment ameliorated the liver pathology. Hepatic steatosis was assessed by liver histology (Figure 1A). On assessing the H&E-stained sections of the liver, Oil Red O staining and the expression level of ChREBP, the levels of lipid accumulation were dramatically increased in the liver of MCD diet mice but were significantly decreased in curcumin-supplemented MCD diet mice. Curcumin exerts beneficial effects against fatty liver disease by reducing hepatic lipid accumulation through the coordination of the AMPK/ACC pathway [38].

Therefore, we assessed liver AMPK regulation by immunoblot assay (Figure 1B). The p-AMPK and p-LKB1 were significantly decreased in the liver tissues of MCD diet mice compared with ND mice. Similarly, AMPK-dependent inhibitory phosphorylation of ACC was increased in MCD diet mice. In contrast, the phosphorylation was recovered in curcumin-supplemented diet mice. ChREBP and SREBP are the essential lipogenic transcription factors involved in the development of hepatic steatosis (Figure 1C). We demonstrated that although the expression of ChREBP and SREBP along with FAS was up-regulated in MCD diet mice when compared with ND mice, curcumin treatment completely attenuated this up-regulated expression.

### 3.2. Curcumin Ameliorates Inflammation Induced in MCD Diet Mice

**Figure 2:**
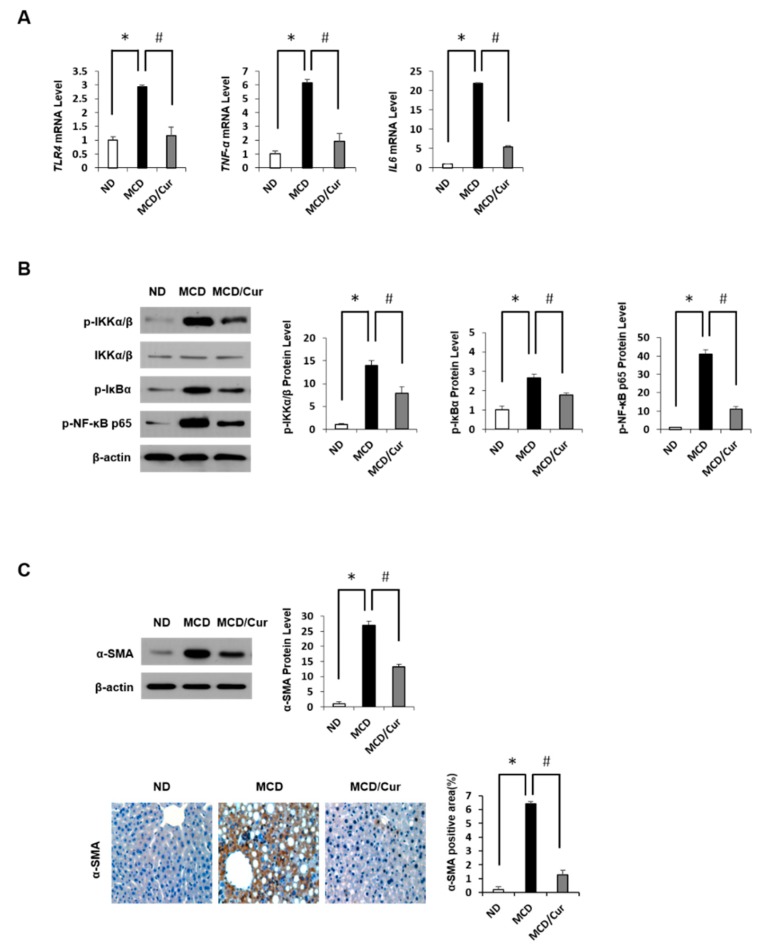
Curcumin modulated the nuclear factor-κB (NF-κB) pathway to alleviate inflammation. Liver tissues were prepared from the mice fed ND, MCD diet, or MCD/curcumin (100 mg/kg) diet. (**A**) The mRNA levels of hepatic Toll-like receptor 4 (*TLR4*), Tumor necrosis factor-alpha (*TNF-α*) and Interleukin 6 (*IL-6*) were measured by quantitative real-time polymerase chain reaction (qRT-PCR). (**B**) Representative immunoblot analyses of phosphorylated IκB kinase α/β (p-IKKα/β), p-IκBα, and p-NF-κB p65. (**C**) Liver sections were subjected to immunoblot and IHC analyses (original magnification 200×) for α-smooth muscle actin (α-SMA). Data are mean ± SD (*n* = 7/group). * *p* < 0.05 vs. ND diet group, and ^#^ *p* < 0.05 vs MCD diet group.

High levels of glucose and lipid are implicated in cellular inflammatory response [39]. To explore proteins involved in inflammation in MCD diet mice, qRT-PCR analysis of liver homogenates was performed (Figure 2A). Levels of *TLR4* mRNA and pro-inflammatory cytokines mRNA, such as *TNF-α* and *IL-6* mRNA, were significantly increased in MCD diet mice when compared with ND mice. However, these increases were significantly reversed by curcumin treatment. NF-κB pathway plays an important role in the response to acute inflammation injury. The active forms of IKK phosphorylate IkBα thus triggers IkBα degradation, resulting in nuclear translocation of NF-κB for activation. The expression of these kinases was significantly up-regulated in MCD diet mice but decreased in curcumin-supplemented MCD diet mice (Figure 2B). During steatohepatitis progression, the increase in the levels of pro-inflammatory cytokines could induce α-SMA, a unique marker for activated hepatic stellate cells and myofibroblasts. immunoblotting and IHC analysis showed that the level of α-SMA was dramatically increased in liver tissues of MCD diet mice, but the expression was significantly decreased in curcumin-treated MCD diet mice (Figure 2C). Taken together, these results revealed that curcumin alleviates inflammation in MCD diet mice by inactivating NF-κB signaling pathway.

### 3.3. DE Analysis of Differentially Expressed Proteins

The proteomes of the mouse liver supplemented with MCD diet or curcumin-supplemented MCD diet were analyzed using two-dimensional gel electrophoresis (2-DE). A typical 2-DE pattern of mouse liver is shown in Figure S2. The tryptic digested peptides from the preparative gel were sequentially subjected to MALDI–TOF MS analysis. Table 1 lists proteins that exhibit not only differential expression with at least a 1.5-fold difference but also those significantly affected by curcumin treatment. The identified thirteen proteins were grouped and classified according to their biological processes. Most of the identified proteins are involved in structural, metabolism and oxidative stress processes. Moreover, the levels of these proteins dramatically changed to a normal level by curcumin-treatment. These results show that curcumin is involved in improving the disturbed metabolic homeostasis in steatohepatitis. Particularly, the eight proteins, marked in blue with an asterisk (*) in Table 1 are known to be susceptible to modification by *O*-GlcNAcylation.

**Figure 3:**
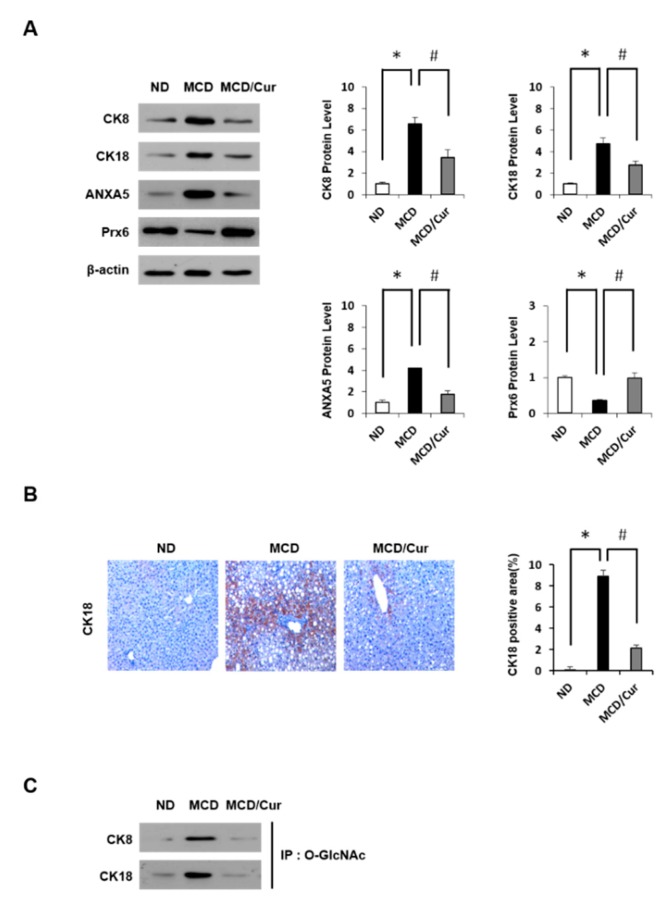
Verification of differentially expressed proteins. Liver tissue extractions were supplied from mice fed ND, MCD diet, or MCD/curcumin (100 mg/kg) diet. (**A**) The hepatic protein expression levels of Cytokeratin 8 (CK8), Cytokeratin 18 (CK18), AnnexinA5 (ANXA5) and peroxiredoxin 6 (Prx6) were determined by immunoblotting using equally identified amounts of total liver protein by immunoblotting. The levels of proteins were normalized to endogenous β-actin protein for each group. Data were obtained three times of independent experiments. (**B**) Liver sections were analyzed by the IHC test with antibody against CK18 (original magnification 200×). (**C**) *O*-GlcNAc (*O*-linked β-*N*-acetylglucosamine) was immunoprecipitated with anti-*O*-GlcNAc antibody and then immunoblotted against CK8 and CK18, separately. Data are mean ± SD (*n* = 7/group). * *p* < 0.05 vs. ND diet group, and ^#^ *p* < 0.05 vs MCD diet group.

To validate our findings from the proteomics approach, immunoblotting analyses and IHC were performed. As shown in Figure 3A, we confirmed that CK8, CK18, and ANXA5 are up-regulated, whereas Prx6 is downregulated in protein expression in MCD diet mice. However, the effects were markedly improved with curcumin treatment, revealing normalization of levels similar to those noted in ND mice, which are consistent with the proteomic results. We also confirmed immunoblot results of CK18 protein by IHC analysis (Figure 3B). In addition, levels of *O*-GlcNAc modification of CK8 and CK18 were further tested using IP against *O*-GlcNAc and immunoblotting analysis (Figure 3C). Interestingly, *O*-GlcNAcylated proteins were up-regulated in MCD diet mice, but this up-regulation was reversed by curcumin treatment. Thus, we focused on dissecting the potential link between HBP and steatohepatitis.

### 3.4. Curcumin Suppresses O-GlcNAcylation in Steatohepatitis

**Figure 4:**
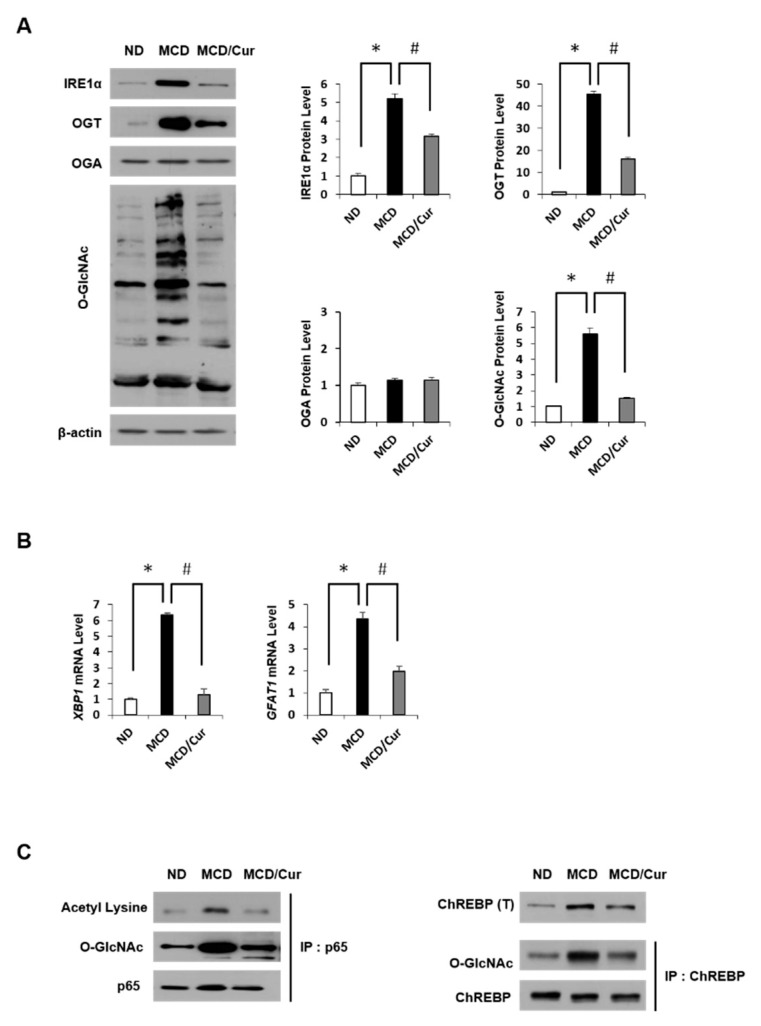
*O*-GlcNAcylation and the proteins associated with HBP. (**A**) Hepatic expression levels of inositol requiring enzyme 1α (IRE1α), *O*-GlcNAc transferase (OGT), *O*-GlcNAcase (OGA) and total *O*-GlcNAcylated proteins by immunoblot analysis in ND, MCD diet, or MCD/curcumin (100 mg/kg) diet mice. In the graphs, the intensity of proteins was determined by densitometry. Data were obtained from three times of independent experiments. (**B**) The levels of protein expression associated with the hexosamine biosynthetic pathway (HBP) were determined by qRT-PCR analysis. The levels of X-box binding protein 1 (*XBP1*) and glutamine:fructose-6-phosphate amidotransferase (*GFAT1*) mRNAs are shown as fold increase. Gene expression levels were normalized to 18s rRNA as an internal standard, and the data are presented as the means ± SD (*n* = 7/group). * *p* < 0.05 vs. ND diet group, and ^#^ *p* < 0.05 vs. MCD diet group. (**C**) The left panel shows that p65 immunoprecipitates were immunoblotted against acetyl lysine and *O*-GlcNAc, separately. Similarly, on the right panel, hepatic expression levels of ChREBP were measured, and ChREBP was also immunoprecipitated and then immunoblotted against *O*-GlcNAc.

In this study, we first investigated the changes of *O*-GlcNAcylation levels in steatohepatitis after curcumin treatment. As shown in Figure 4A, MCD diet mice showed not only the induction of *O*-GlcNAcylation but also the up-regulation of OGT and IRE1α expression. All these levels decreased dramatically in response to curcumin treatment. Conversely, OGA expression was not significantly affected by any treatment. In addition, we examined the expression level of HBP-related proteins through mRNA levels. In Figure 4B, the mRNA levels of *XBP1* were significantly upregulated in MCD diet mice, but the levels dramatically decreased in curcumin-supplemented MCD diet mice. Similar results were obtained for GFAT. Furthermore, we tested how *O*-GlcNAcylation is involved in the expression of NF-κB p65 or ChREBP in ER stress and lipid accumulation, further estimating the role of curcumin. To define the connection of *O*-GlcNAcylation and acetylation in the activation of p65, IP analysis with p65 antibody was performed, followed by immunoblotting using the *O*-GlcNAc antibody (Figure 4C, left panel). The degree of *O*-GlcNAcylation was increased in MCD diet mice but showed a significantly suppressed in curcumin-supplemented MCD diet mice. Interestingly, the acetylation along with the expression level of NF-κB p65 was increased in MCD diet mice. However, this increase was reversed by curcumin treatment. We also observed an increase in ChREBP expression levels in MCD diet mice (right panel). Similarly, the *O*-GlcNAc modification to ChREBP was upregulated in MCD diet mice, but treatment with curcumin significantly reduced the effect. These results revealed that curcumin could reduce hepatic levels of OGT and GFAT expression in MCD diet mice, which correlated to the decrease in *O*-GlcNAcylated protein levels of steatohepatitis.

### 3.5. Regulation of Antioxidant Proteins in Palmitate-Induced AML12 Cells

Intracellular accumulation of lipids is a hallmark of hepatosteatosis, which may lead to oxidative stress. Thus, in parallel with the liver tissue data, we used a mouse hepatic cell line AML12 cells to explore the association of *O*-GlcNAcylation regulation with antioxidant protein expression during oxidative stress. Lipid accumulation in AML12 cells treated with palmitate for 24 h was significantly increased, but lipid accumulation in curcumin-treated cells treated with palmitate was lowered to normal (Figure S3A). Intracellular lipid droplet accumulation was confirmed via Oil Red O staining.

**Figure 5:**
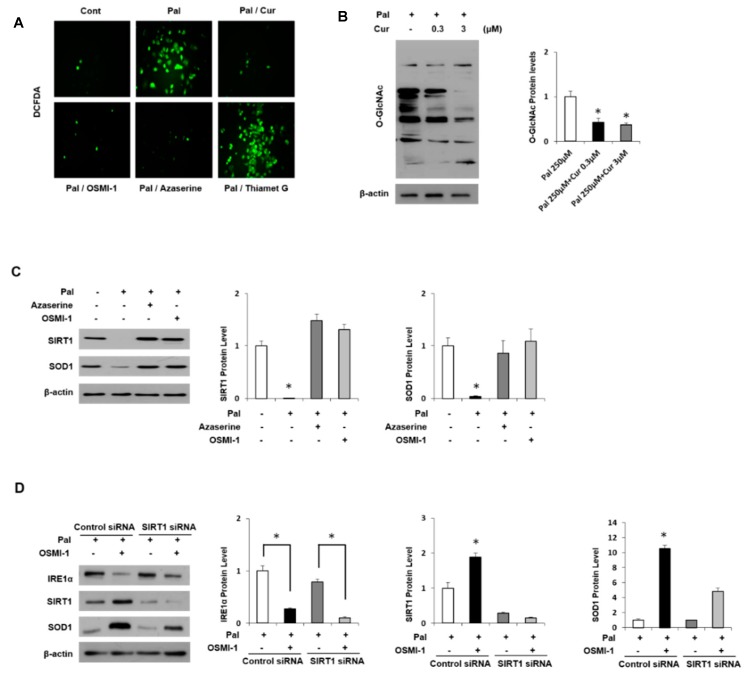
Regulation of *O*-GlcNAcylation and antioxidant protein in AML12 cells. (**A**) AML12 cells were either untreated or pretreated with 250 μM palmitate (Pal) and incubated with curcumin (3 μM), OSMI-1 (20 μM), Azaserine (100 μM) or Thiamet-G (200 μM) for 12 h. The levels of ROS in cells were measured via DCFDA fluorescence. (**B**) AML12 cells were treated with Pal (250 μM) and different concentrations of curcumin (0.3 or 3 μM) to analyze the level of *O*-GlcNAcylated proteins by immunoblot assay. (**C**) The cells were either untreated or pretreated with 250 μM Pal and incubated with Azaserine (100 μM) or OSMI-1 (20 μM) for 12h. Levels of SIRT1 and SOD1 expression were measured by immunoblot assay. (**D**) AML12 cells were transfected with control or SIRT1 siRNA for 24 h, and then the cells were treated with or without OSMI-1 (20 μM), followed by incubation with 250 μM Pal for 12 h. Levels of IRE1α, SIRT1, and SOD1 expression were measured by immunoblot analysis. All expression levels were normalized relative to β-actin. The data from three independent experiments are presented as the means ± SD. * *p* < 0.05.

Interestingly, treatment with OSMI-1 and Azaserine, inhibitors of OGT and GFAT, respectively, significantly prevented lipid deposition, whereas treatment with the OGA inhibitor Thiamet-G maintained *O*-GlcNAcylation levels and resulted in lipid accumulation in AML12 cells. In addition, DCFDA analysis was used to examine intracellular ROS levels (Figure 5A). Owing to lipid accumulation, intracellular ROS levels were significantly increased in palmitate-treated AML12 cells but decreased by curcumin treatment and regulated by the level of *O*-GlcNAcylation. Similarly, inhibition of OGT or GFAT decreased ROS levels, whereas treatment with the OGA inhibitor did not affect intracellular ROS levels. Thus, curcumin suggests lowering lipid accumulation and ROS levels through mechanisms that inhibit *O*-GlcNAcylation signaling.

The palmitate-induced oxidative stress level decreased the *O*-GlcNAcylation level in a curcumin dose-dependent manner (Figure 5B). Changes in *O*-GlcNAcylation and the related regulatory proteins in oxidative stress induced by palmitate were identified. As shown in Figure S3B, the levels of *O*-GlcNAcylation protein and the expression levels of IRE1α and OGT, regulatory proteins, were increased dose-dependently in response to palmitate treatment. In contrast, the change in the expression of OGA did not change significantly, which is consistent with the results observed in MCD diet mice. Interestingly, the expression level of SIRT1 was inversely related to *O*-GlcNAcylation and OGT levels. Recent studies in this regard have indicated that the induction of *O*-GlcNAcylation or elevated levels of OGT down-regulates SIRT1 levels and activity [40]. Similarly, the inhibition of OGT expression and activity in breast cancer cell lines increased SIRT1 [41]. We further investigated the role of SIRT1 and *O*-GlcNAcylation in an antioxidant protein we identified, which is mentioned in Table 1. It is well known that SOD1 is a major enzyme antioxidant, which protects cells from ROS-induced damage. In AML12 cells, SOD1 was positively regulated by SIRT1, and palmitate treatment significantly decreased SIRT1 expression and thereby blocked SOD1 expression (Figure 5C). However, treatment with GFAT inhibitor or OGT inhibitor increased their expression and eventually normalized it. In addition, the blocking of *O*-GlcNAcylation signal by OSMI-1 treatment increased SIRT1 expression and thereby increased SOD1 expression (Figure 5D). Conversely, when SIRT1 was knocked down, the expression of SOD1 was not increased although the expression of upper IRE1α was reduced by OSMI-1 treatment. Similar experiments were carried out with curcumin instead of the OGT inhibitor (Figure S3C). ER stress induced by palmitate stimulation is dramatically relieved by curcumin treatment, which increased SIRT1 and SOD1 expression, but the siRNA SIRT1 treatment blocked SOD1 expression regardless of *O*-GlcNAcylation blocking. These results show that *O*-GlcNAcylation signals and curcumin are associated with SIRT1 expression and consequently regulated SOD1 expression.

### 3.6. Curcumin Ameliorates Inflammation by Inhibiting of O-GlcNAcylation and Activates Antioxidant Protein

**Figure 6:**
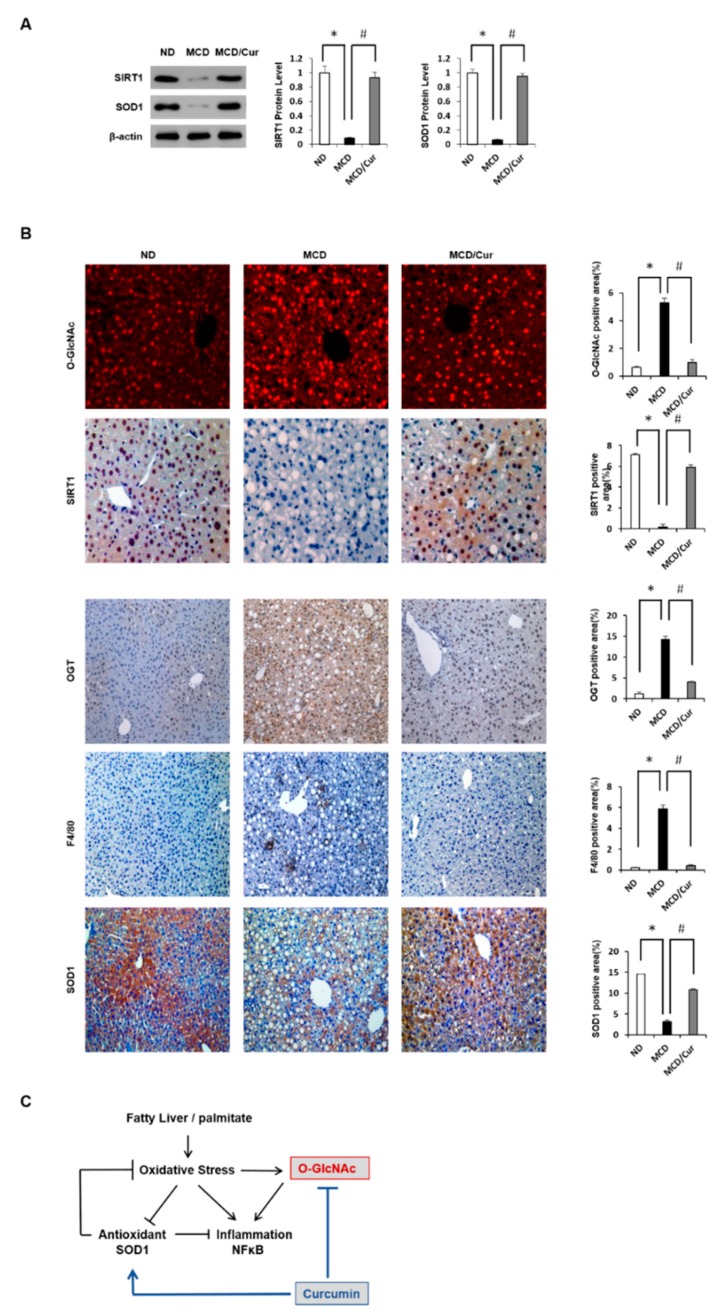
Curcumin prevents inflammation by inhibiting *O*-GlcNAcylation and activates antioxidant proteins. (**A**) Liver lysate was prepared from mice fed with ND, MCD diet, or MCD/curcumin (100 mg/kg) diet. Hepatic expression levels of SIRT1 or SOD1 were measured by immunoblot analysis. The levels of proteins were normalized to endogenous β-actin protein for each group. The relative amounts of the proteins are expressed as fold-increases. Data were obtained from three times of independent experiments. * *p* < 0.05 vs. ND diet group, and # *p* < 0.05 vs. MCD diet group. (**B**) IHC analysis of *O*-GlcNAcylation, SIRT1 (original magnification 400×), OGT, F4/80, or SOD1 (original magnification 200×) in livers of ND, MCD diet, or MCD/curcumin diet mice. Data are mean ± SD (n = 7/group). * *p* < 0.05 vs. ND diet group, and # *p* < 0.05 vs. MCD diet group. (**C**) The proposed representation showed that curcumin up-regulates antioxidant expression but down-regulates the NF-κB pathway by blocking *O*-GlcNAcylation signaling pathway. Curcumin-induced SOD1 down-regulates ROS stress and inflammatory signaling. Arrow and bar represent activation and suppression, respectively.

We demonstrated again in the liver tissues that SIRT1 positively regulates the expression of an antioxidant protein SOD1. Immunoblot showed that protein expression of SIRT1 and SOD1 was significantly down-regulated in MCD diet mice, but the effects were significantly improved in curcumin-supplemented MCD diet mice to levels comparable to those in ND mice (Figure 6A).

In addition, IHC analysis confirmed the immunoblot results of *O*-GlcNAc regulation and SIRT1 protein acting on inflammatory mechanisms (Figure 6B). *O*-GlcNAc-modified proteins were increased in MCD diet mice compared to ND mice. Conversely, SIRT1 expression levels decreased in MCD diet mice but increased again in curcumin-treated mice to levels comparable to those noted in ND mice. Similarly, OGT expression in MCD diet mice was up-regulated compared to those in ND and curcumin-supplemented MCD diet mice. Antibodies against F4/80, a marker of mature macrophages, were used to characterize NF-κB-induced inflammation accumulated in the liver in MCD diet mice. Thus, we confirmed increased hepatic macrophage accumulation in MCD diet mice but decreased in the curcumin-supplemented MCD diet mice. In addition, the IHC analysis for SOD1 showed the same results as the immunoblot shown in Figure 6A. Taken together, as shown in Figure 6C, oxidative stress induced by lipid accumulation in liver cells activated NF-κB inflammatory signals but inhibited antioxidant pathways. In contrast, the *O*-GlcNAcylation pathway positively regulated to the inflammatory signaling but indirectly and negatively regulated to the antioxidant process. Curcumin is known to reduce intracellular inflammatory activity and enhance antioxidant effects. In conclusion, we proposed that curcumin blocks the *O*-GlcNAcylation pathway, thereby alleviating the toxicity of lipid accumulation.

## 4. Discussion

Curcumin is well known to protect against the pathologic effects of obesity and related metabolic disorders. In this study, we confirmed that curcumin suppresses fat accumulation and inflammation in hepatocytes while activating antioxidant mechanisms. To identify proteins that are differentially expressed in hepatitis mice as well as have an improved effect on curcumin treatment, a proteomics approach was used. More interestingly, eight of the identified proteins were known to undergo *O*-GlcNAcylation modification. The *O*-GlcNAcylation level is known to be up-regulated in NASH mice. Therefore, we mainly focused on how the mechanism of *O*-GlcNAcylation induced by steatohepatitis is associated with the alleviation of inflammation by curcumin treatment.

A causative link between the pathology of NASH and the up-regulation of *O*-GlcNAcylation is not yet clear. However, excess lipid accumulation in tissues is closely related to increased fatty acid oxidation due to ER stress and activation of HBP via ROS. A recent study reported that GFAT is transcriptionally up-regulated under ER stress, and this up-regulation leads to increased protein *O*-GlcNAcylation and provides a protective effect against liver injury [42]. Here, we showed that *O*-GlcNAcylation was increased in MCD diet mice, and OGT and GFAT were also up-regulated. In addition, the upstream target IRE1α induced by ER stress resulted in the up-regulation of OGT and GFAT in MCD diet mice. These results suggested that the induction of OGT and GFAT by the transcriptional regulation of XBP1 as an upstream activator of HBP activates a positive regulatory loop in NASH pathogenesis. Curcumin effectively suppressed protein *O*-GlcNAc modification and further down-regulated ER stress by inhibiting XBP-associated IRE1α and GFAT expression. In conclusion, these results suggested that curcumin alleviates hepatitis by blocking HBP flux signaling. To our knowledge, this is the first report demonstrating the down-regulation of HBP by curcumin in steatohepatitis.

In parallel with lipid accumulation, *O*-GlcNAcylation was significantly increased in NAFLD [43]. An increase in *O*-GlcNAcylation by FFA and glucose has been reported in hepatocytes and carcinoma cell lines [44]. In the present study, *O*-GlcNAcylation was increased by FFA in AML12 cells, while it was clearly demonstrated that OGT was down-regulated under curcumin treatment conditions, resulting in reduced *O*-GlcNAcylation. The effect of curcumin on fat accumulation and inflammation in liver tissue has been studied previously [45]. Thus, we confirmed that curcumin upregulates the expression of phosphorylated AMPK, presumably via LKB1 [38]. In previous studies, *O*-GlcNAcylation reportedly increased the expression of ChREBP, which increases the transcriptional activity of FAS and ACC genes, resulting in excessive TG deposition in the liver [46]. In the present study, we observed the induction of *O*-GlcNAcylated ChREBP in MCD diet mice but significantly reduced *O*-GlcNAcylation levels in curcumin-supplemented MCD diet mice. Hepatic SREBP and FAS were also dramatically decreased by curcumin, while MCD induced lipogenesis was upregulated along with *O*-GlcNAcylation. Together, these data suggest that increased levels of lipogenesis in MCD diet mice led to increased ROS levels, which can lead to HBP flux causing the up-regulation of the *O*-GlcNAcylation process. On curcumin treatment, *O*-GlcNAcylation regulation is inhibited along with the action of lowering ROS levels, resulting in improved lipid accumulation.

Previous studies have shown a link between chronic inflammation and hepatic steatosis. The response to inflammatory damage in animal models of obesity is mainly via the NF-κB pathway [47]. The NF-κB dimer comprises p65 (RelA) and p50 subunits, where the *O*-GlcNAcylated p65 is released from the isolation by IκB, resulting in an increase of nuclear translocation of NF-κB. Consequently, up-regulated *O*-GlcNAcylation contributes to NF-κB activation and exacerbates inflammatory damage [48]. In this study, we biochemically demonstrated that curcumin alleviates inflammation by inhibiting TNFα and IL6 production and blocking the NF-κB pathway. *O*-GlcNAcylation of RelA/p65 was up-regulated in MCD animal models, while OGT inhibition blocked nuclear translocation of NF-κB p65 and alleviated inflammation. Interestingly, we obtained similar results in the treatment of curcumin on behalf of OGT inhibitors. Thus, curcumin appears to inhibit NF-κB activity by blocking the *O*-GlcNAcylation process, which is essential for the translocation of NF-κB. In addition, we showed that when RelA/p65 is *O*-GlcNAcylated in MCD diet mice, the acetylation level was increased but the deacetylation factor SIRT1 was decreased. SIRT1 participates in the inflammatory response by deacetylating NF-κB, particularly the p65 subunit. Curcumin as a SIRT1 activator reportedly inhibits the up-regulation of inflammatory markers [49]. Although further experiments are needed to elucidate the regulatory mechanism, it is clear that *O*-GlcNAcylation levels are inversely proportional to SIRT1 expression in the presence of fat accumulation. Consequently, curcumin administration induces these PTM regulations of the NF-κB pathway, presumably reducing the production of IL-6 and TNF- α and eventually suppressing hepatitis.

ROS levels in the tissues of NASH-affected liver are increased, whereas the expression levels of antioxidant enzymes are decreased [50]. Curcumin is well known to not only attenuate lipid peroxidation and oxidative stress but also enhance antioxidant systems, including SOD1, GPx, and CAT [51]. SIRT1 is extensively studied and has a significant role in metabolic organs, such as the liver, adipose, and skeletal muscle tissues. Recent studies have indicated that SIRT1 upregulation in the liver injury protects from lipid-induced hepatic inflammation by SOD1, thus increase its expression. A recent study reported that the inhibition of OGT expression and activity in breast cancer cell lines could induce SIRT1 stability. In mouse models, curcumin reduced ER stress in adipose tissues, which showed a significant increase in the expression of SIRT1. In the present study, we investigated how *O*-GlcNAcylation and SIRT1 regulation are involved in SOD1 expression in AML12 cells and MCD diet mice. Treatment with OGT inhibitors in palmitate-induced AML12 cells confirmed that SOD1 levels increased as *O*-GlcNAcylation decreased. Palmitate is known to induce ROS production, which is one of the main causes of inflammation and cellular stress, and induce steatosis by activating several fat-forming enzymes, such as ACC and FAS [52]. Conversely, cells treated with SIRT1 siRNA did not increase the expression of SOD1 even when OGT was inhibited. These results suggest that SIRT1 is positively regulated SOD1 expression but negatively regulates *O*-GlcNAcylation. This suggests that the regulation of *O*-GlcNAcylation and SOD1 expression are inversely related to each other. In this study, we also found that treatment with OGT inhibitors inhibited the production of PA-induced ROS and that this effect was similar to the results of curcumin treatment (Figure 5A). These results were also confirmed in MCD diet mice, in which the expressions of SOD1 and SIRT1 were significantly down-regulated, while these effects were dramatically recovered by curcumin treatment (Figure 6A). Taken together, these results revealed that curcumin can enhance antioxidant enzyme activity by negative regulation of HBP. Thus, curcumin contributes to SOD1, which is a key factor in the regulation of inflammation and antioxidant defense systems, which prevents the hepatotoxicity of MCD.

In summary, oxidative stress from NASH induces NF-κB inflammatory signals and antioxidant pathways that are inversely controlled with each other. In this study, we identified antioxidant proteins such as SOD1, GPx, and Prx6, which are differentially expressed in MCD diet mice but up-regulated by curcumin treatment. Moreover, we also demonstrated that the regulation of *O*-GlcNAcylation is a key mechanism by which curcumin resolves the imbalance of lipid metabolism. In addition, we have shown that the anti-inflammatory influence of curcumin was mediated by blocking the NF-κB signaling pathway by inhibiting *O*-GlcNAcylation. To our knowledge, this is the first report that curcumin inhibits the *O*-GlcNAcylation process for exerting anti-inflammatory effects in NASH mice. The up-regulation of *O*-GlcNAcylation pathway by oxidative stress appeared to be associated with inflammatory signaling, but it had a negative effect on antioxidant activity. In conclusion, we suggested that curcumin alleviates the lipid toxicity by blocking the *O*-GlcNAcylation pathway and by increasing antioxidant proteins. The next step should be to assess the molecular mechanisms involved in the regulation of *O*-GlcNAcylation of antioxidant proteins identified in this work. *O*-GlcNAcylation studies suggest a new aspect of pathogenesis in NASH and appear to be used as potential biomarkers in the near future.

## Figures and Tables

**Table 1:** Differentially expressed proteins list.

No ^a^	Identified Protein	Accession No.	MW (KDa)	PI	MOWSE Score	Coverage (%)	Fold Change	
							MCD	MCD + Cur
**Metabolism**								
1	14-3-3 protein zeta/delta	P63101	27.8	4.7	30,049	44.9	4	3.2
2	Annexin A5	P48036	35.8	4.8	4.29 × 10^8^	47.3	6	3.5
3	2’-5’-oligoadenylate synthase-like protein 1	Q8VI94	59.1	6.7	139,169	22.9	3	1
Transport								
4	40S ribosomal protein SA *	P14206	32.8	4.8	21,692	40.3	3	1
5	Integrin beta-like protein 1	Q8VDV0	54.0	5.3	14,545	20.0	2	0.8
Oxidative Stress/Detoxification processes								
6/7	AOP2(Peroxiredoxin-6) *	O08709	26.3	6.2	2350	18.5	0.4	1
8	Glutathione peroxidase 1 (GPx 1) *	P11352	22.3	6.7	141,506	42.3	0.6	1.3
9	SODC(Superoxide dismutase [Cu-Zn]) *	P08228	15.9	6.0	1996	41.6	0.3	0.8
10	60 kDa heat shock protein, mitochondrial *	P63038	61.0	5.9	1.60 × 10^6^	43.8	2	1
Structural Proteins								
11/12	Keratin, type I cytoskeletal 18 *	P05784	47.5	5.2	3.72 × 10^7^	36.6	5	1.5
13/14/15	Keratin, type II cytoskeletal 8 *	P11679	54.6	5.7	1.25 ×10^7^	42.0	3	1
16	Actin-related protein T1	Q9D9J3	42.2	5.2	701	18.6	5	3
17	Rho GDP-dissociation inhibitor 1 *	Q99PT1	23.4	5.1	2545	41.7	4	2.5

^a^ The identified protein numbers correspond to the 2-DE gel numbers in Figure S2. * indicates *O*-GlcNAc modifiable protein.

## Supplementary Materials

The following are available online at https://www.mdpi.com/2072-6643/11/11/2702/s1, Figure S1: ND, MCD diet, and MCD/curcumin diet mice. Mice were fed ND, MCD diet, or MCD/curcumin diet for 3 weeks., Figure S2: Protein expression map of mouse liver. Coomassie Blue-stained 2DE gel shows proteins derived from mice fed ND, MCD diet or MCD/curcumin diet., Figure S3: Effect of palmitate on lipid accumulation, *O*-glcNAcylation and SIRT1 siRNA in AML12 cells.
