# Supplementary material for: Curcumin Ameliorates Nonalcoholic Fatty Liver Disease through Inhibition of O-GlcNAcylation
Source: Nutrients. 2019 Nov 8;11(11):2702. doi: 10.3390/nu11112702 (PMC6893521; doi:10.3390/nu11112702)
Supplement: Supplementary file 1 [file nutrients-11-02702-s001.pdf]

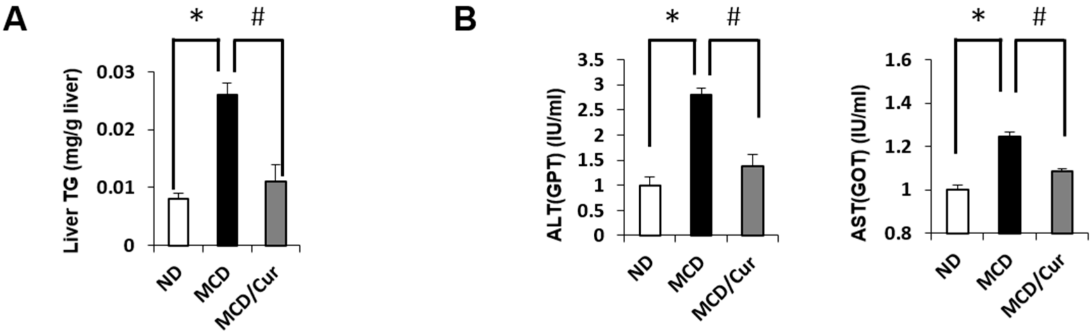

Figure S1. ND, MCD diet, and curcumin + MCD diet-fed mice. Mice were fed ND, MCD diet, or MCD + curcumin diet for 3 weeks. (**A**) Body weight and liver TG levels, and (**B**) serum ALT and AST levels were measured. Data are mean ± SD (n = 7/group). * $p < 0.05$ vs. ND group and # $p < 0.05$ vs. MCD group.

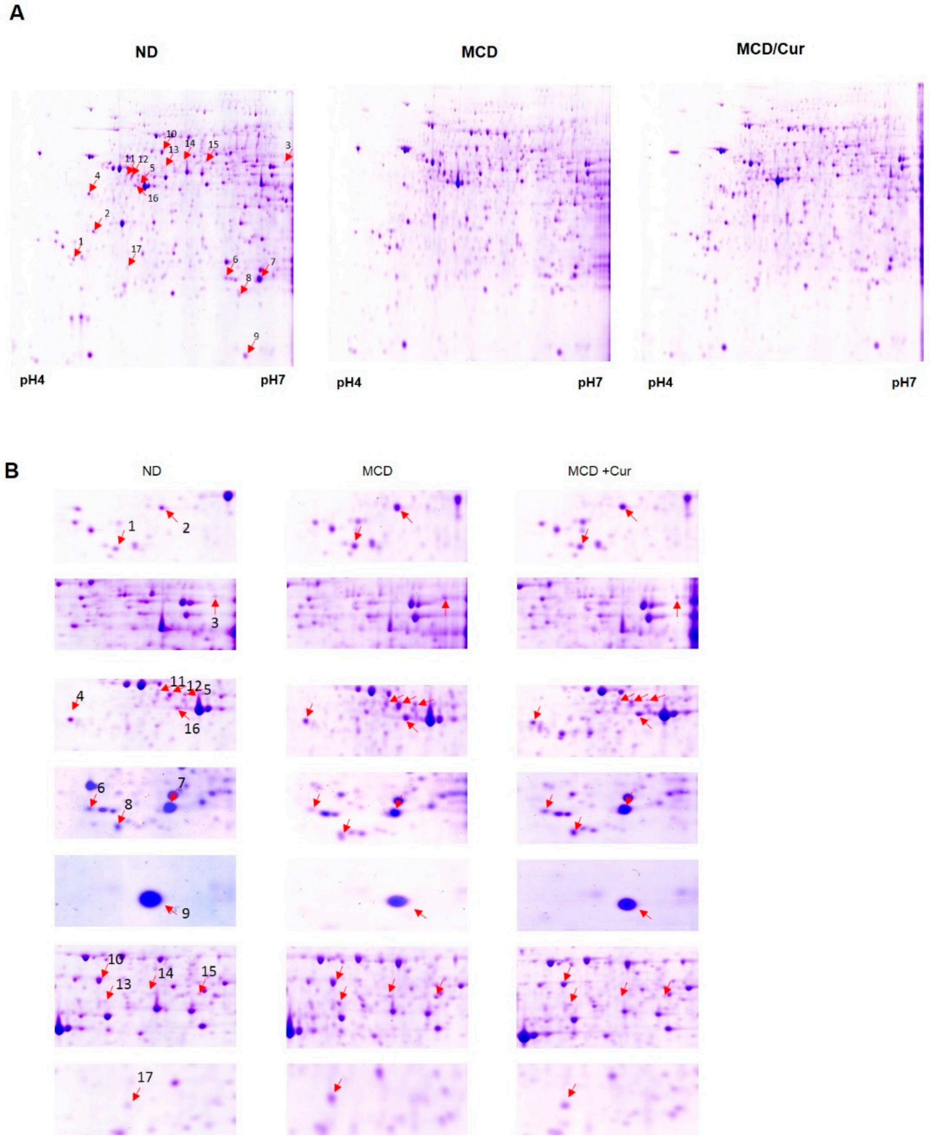

Figure S2. Protein expression map of mouse liver. Coomassie Blue-stained 2DE gel shows proteins derived from mice fed ND, MCD diet or MCD + curcumin diet. The proteins from mouse liver were loaded on a 24 IPG strip (pH 4–7) and then run on SDS-PAGE (12%). The protein spots significantly affected by different diet feeding are indicated by arrows. (**A**) and (**B**) The numbers on the gel correspond to the spot numbers in Table 1.

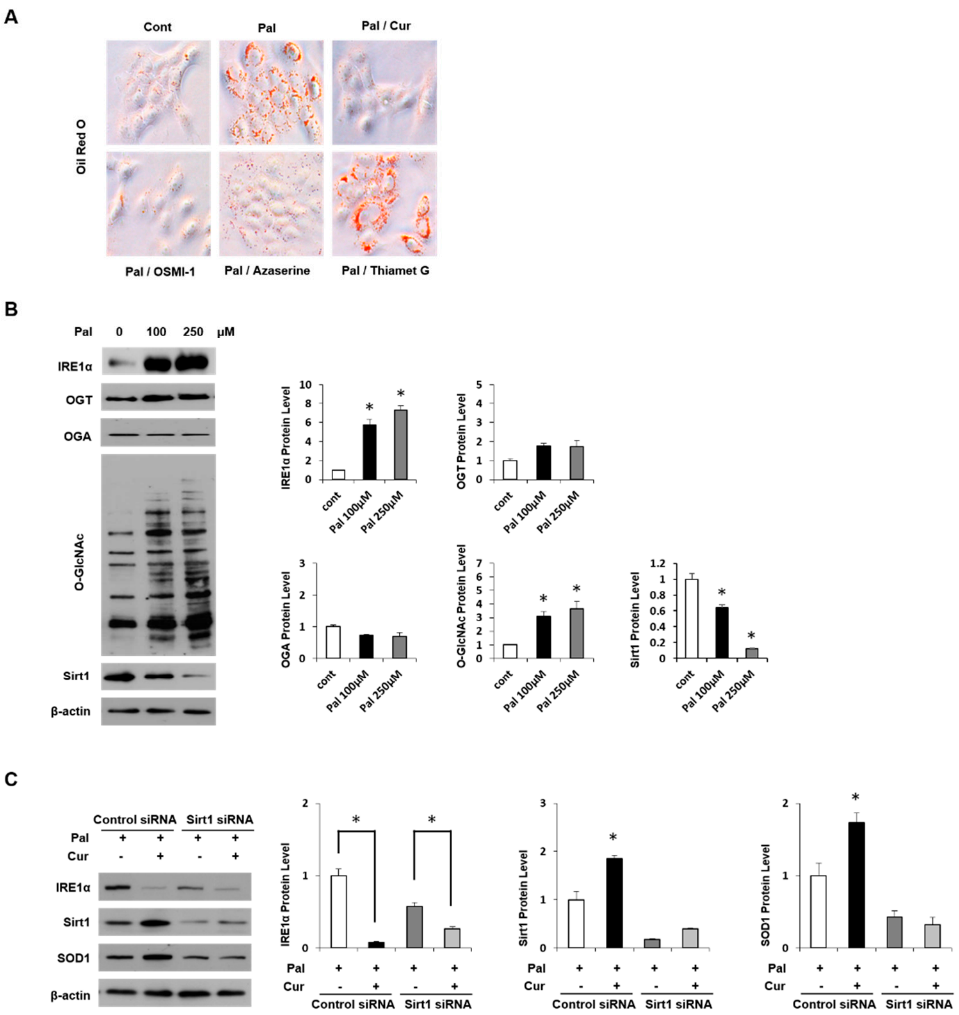

Figure S3. Effect of palmitate on lipid accumulation, *O*-glcNAcylation and SIRT1 siRNA in AML12 cells.(**A**) AML12 cells were either untreated or pretreated with 250 μM palmitic acid and incubated with curcumin, OSMI-1(20 μM), azaserine (100 μM) or Thiamet G (200 μM) for 12 h. Lipid accumulation in the cells was measured by Oil Red O staining. (**B**) The cells were not treated or pretreated with 100 or 250 μM palmitic acid for 12h. The expression levels of *O*-GlcNAcylated proteins, such as IRE1*α*, OGT, OGA, and SIRT1, were measured by immunoblot analysis. The data were presented as the means of ± SD. Of three independent experiments. * $p < 0.05$. (**C**) Cells were transfected with control or SIRT1 siRNA after 24h and then treated with 250 μM palmitic acid with or without curcumin (3 μM) for 12 h. Levels of IRE1*α*, SIRT1, and SOD expression were measured by immuneblot analysis. Expression levels were normalized relative to β-actin. The data from three independent experiments were presented as the means ± SD. * $p < 0.05$.
